# Higher efficacy of rupatadine 20 mg and 10 mg versus placebo in patients with perennial allergic rhinitis: a pooled responder analysis

**DOI:** 10.1186/s13223-020-00425-1

**Published:** 2020-04-23

**Authors:** Antonio Valero, Iñaki Izquierdo, Marek L. Kowalski, Glenis K. Scadding, Jean Bousquet, Joaquim Mullol

**Affiliations:** 1 Allergy Section, Pneumology and Allergy Department, Hospital Clínic de Barcelona, Institut d’Investigacions Biomèdiques August Pi i Sunyer (IDIBAPS), Barcelona, Catalonia Spain; 2 CIBER de Enfermedades Respiratorias (CIBERES), Barcelona, Catalonia Spain; 3 Department of Clinical Development & Medical Adviser, Biohorm, Grupo Uriach, Avinguda Camí Reial, 51-57, 08184 Barcelona, Catalonia Spain; 4 Department of Immunology and Allergy, Medical University of Lodz, Lodz, Poland; 5 Department of Allergy and Rhinology, Royal National Ear, Nose and Throat Hospital, London, UK; 6 MACVIA-France, Contre les Maladies Chroniques Pour un VIeillissement Actif en France European Innovation Partnership on Active and Healthy Ageing Reference Site, Montpellier, France; 7 Rhinology Unit & Smell Clinic, ENT Department, Hospital Clínic de Barcelona, Institut d’Investigacions Biomèdiques August Pi i Sunyer (IDIBAPS), Universitat de Barcelona, C/Villarroel, 170, 08036 Barcelona, Catalonia Spain

**Keywords:** PAF antagonist, Perennial allergic rhinitis, Responder analysis, Rupatadine, Second-generation H_1_-antihistamines

## Abstract

**Background:**

The clinical efficacy of rupatadine in terms of responders has not been previously explored in perennial allergic rhinitis (PAR).

**Methods:**

This pooled analysis included data from 6 randomised, double-blind, placebo-controlled trials conducted in PAR patients treated with rupatadine 10 mg or 20 mg, or placebo. Participants were aged ≥ 18 years, with diagnosis of PAR and a Total 4 Nasal Symptom Score (T4NSS) ≥ 5. We evaluated the T4NSS and Total 5 Symptom Score (T5SS) for 28 days of treatment, the responder proportion (50% and 75% response), and the time to response.

**Results:**

Efficacy data from 1486 patients were analysed: 585 received placebo, 682 rupatadine 10 mg, and 219 rupatadine 20 mg. Compared with placebo, rupatadine promoted greater symptom improvements and higher responder proportions (50% and 75% response) for T4NSS and T5SS over 28 days. Symptom improvements and responder proportions were higher in the rupatadine 20 mg group vs the 10 mg group. The time to response was shorter in the rupatadine 20 mg group vs the 10 mg group for T4NSS (16 and 9 days for the 50% and 75% responses, respectively) and for T5SS (13 and 8 days for the 50% and 75% responses, respectively).

**Conclusions:**

Rupatadine was efficacious in reducing allergic rhinitis symptoms, showing high responder proportions. The faster and stronger effect of rupatadine 20 mg may suggest its use in patients with severe PAR or not responding to the standard dose.

## Background

Allergic rhinitis (AR), an inflammatory disorder of the nasal mucosa, is a prevalent condition and a significant public health problem [1]. The prevalence of AR varies widely among countries, affecting 10–40% of the population worldwide and currently rising [2].

Patients with AR experience nasal itching, sneezing, rhinorrhea, and nasal congestion, whereas ocular symptoms such as tearing, eye itching, and redness are also common. Although AR is not a life-threatening condition, clinical manifestations result in fatigue, sleep disturbance, and reduced work/school productivity, severely impairing quality of life [3, 4]. Besides, due to its prevalence and chronicity, AR is associated with significant healthcare costs [5, 6].

Classically, AR has been classified by the duration of exposure and type of allergens into seasonal AR (SAR) and perennial AR (PAR). In PAR, the allergens are present year-round and mainly include dust mites, insects and pets with fur [7]. Nasal congestion and rhinorrhea are the predominant symptoms in PAR patients, and they have a significantly higher incidence of moderate-to-severe AR [8] and degree of interference of the disease in daily life activities or sleep compared with SAR [9, 10]. The Allergic Rhinitis and its Impact on Asthma (ARIA) guideline proposed a newer classification in 2001, which was updated in 2008, based on the duration of AR symptoms, comprising two broad groups: intermittent and persistent AR [11, 12].

Clinical manifestations of AR are the result of a complex cascade, in which the contact of the external trigger with the nasal mucose leads to the release and degranulation of inflammatory mediators [13]. A wide range of inflammatory cells is involved in this IgE-mediated response, being histamine one of the major contributors to hallmark AR symptoms. The platelet-activating factor (PAF) was later discovered as a mediator of nasal congestion and rhinorrhea symptoms of AR since it promotes an increase in vascular permeability and bronchoconstriction [14].

Second-generation H_1_-antihistamines are currently recommended as first-line treatment for patients with PAR because of their proven efficacy and safety with minimal sedating effects [2, 15]. Given the important role of histamine and PAF on the allergic response, H_1_-antihistamines targeting both determinants could be advisable therapeutic approaches. Current guidelines also recommend treatments with broad activity such as intranasal corticosteroids, the intranasal formulation of azelastine and fluticasone propionate (MP-AzeFlu) or allergen immunotherapy (AIT) for the treatment of allergic rhinitis in patients whose symptoms are not well controlled [2, 12, 15].

Rupatadine (Uriach and Cía, Barcelona, Spain) is a second-generation H_1_-antihistamine with a dual mechanism of action targeting both histamine and PAF. This H_1_-antihistamine is currently indicated for the treatment of SAR, PAR and chronic urticaria in adults > 12 years and children [16]. The superior efficacy of rupatadine vs placebo was previously demonstrated in several randomised, controlled trials [17–21]. In patients with PAR, rupatadine was not inferior to ebastine [22], loratadine [23] and cetirizine [24, 25].

Although the superior efficacy of rupatadine vs placebo has been largely demonstrated, yet there is an uncovered need to address the clinical relevance of rupatadine response in PAR. The European Medicines Agency (EMA) guideline on the treatment of allergic rhinoconjunctivitis recommends assessing the proportion of responders (≥ 50% reduction in symptoms) to determine the clinical effect of AR treatments [26].

We have recently shown, through a responder analysis, that rupatadine 10 mg, the standard dose, and rupatadine 20 mg, with higher and faster efficacy, promoted a clinically relevant effect in SAR patients [27]. In the present study, we pooled data of randomised clinical trials in patients with PAR treated with rupatadine and assessed the proportion of responders by the criteria described previously [27].

## Methods

### Study design

This study was a pooled analysis of data from 6 randomised, double-blind, placebo-controlled clinical trials conducted in patients with PAR treated with rupatadine 10 mg or 20 mg, or placebo [22–25, 28].

The studies included complied with the declaration of Helsinki and ICH Guidelines on Medicinal products and obtained previous ethical approval. All patients provided written informed consent before the inclusion in each study [22–25, 28].

In this post hoc analysis, efficacy data were pooled and analysed according to the criteria previously described for SAR [27]. Data analysed comprised patients’ daily self-recordings of symptoms at baseline and upon treatment with rupatadine 10 mg or 20 mg, or placebo. Rupatadine or placebo were administered once a day in the morning as oral tablets of identical appearance by their encapsulation in gelatine capsules (Capsugel^®^). Although 2 studies were designed with longer treatment duration, in this pooled analysis, the first 28 days of treatment with rupatadine (10 or 20 mg) or placebo were analysed (see Additional file 1: Table S1).

### Study population

The studies included men or women aged 18 years or older, with diagnosis of PAR for at least 12 months before inclusion and with a Total 4 Nasal Symptom Score (T4NSS) ≥ 5 at baseline [22–25, 28].

Patients with non-allergic rhinitis (vasomotor, infectious, drug-induced) were excluded, as were those with other conditions that could have interfered with the treatment such as chronic rhinosinusitis (CRS), with or without nasal polyps, or a significant deviation of the nasal septum. Other exclusion criteria included concomitant treatment with specific medications such as nasal decongestants within the previous 24 h, topical antihistamines within the previous 48 h, oral antihistamines or disodium cromoglycate within the previous week, and systemic or intranasal treatment with corticosteroids or immunosuppressants within the previous 2 weeks [22–25, 28].

### Study outcomes

#### Change in symptom score

We evaluated the change in symptom severity from baseline over a 28-day treatment period in rupatadine and placebo groups.

Symptoms were grouped into the T4NSS and Total 5 Symptom Score (T5SS). The T4NSS comprises rhinorrhea, nasal congestion, nasal itching, and sneezing, whereas the T5SS also includes ocular itching. Each symptom is graded on a 4-point scale (0 = absent, 1 = mild, 2 = moderate, and 3 = severe) resulting in a maximum score of 12 for the T4NSS and of 15 for the T5SS.

#### Responder analysis

The 50% and 75% response was defined as a significant reduction in symptom scores ≥ 50% or ≥ 75% (for either T4NSS or T5SS), respectively. We assessed the proportion of responders for each response cut-off on days 1, 7, 14, 21, and 28.

#### Time to response

This outcome defines the time needed to achieve a proportion of responders for the 50% or 75% response. The time to achieve a 50% proportion of responders for the 50% response and the time to reach a 25% proportion of responders for the 75% response was compared between treatment groups for the T4NSS or T5SS.

### Statistical analyses

Continuous variables were defined as mean and standard deviation (SD) and categorical variables as number and percentage.

Comparisons between treatment groups were performed using the non-parametric Kruskal–Wallis test followed by the Mann–Whitney test for symptom score evolution and the Chi-square test for the proportion of responders.

The SAS software (SAS Institute, Cary, SC, USA) for Windows, version 9.2, was employed for statistical analyses. A *p *< 0.05 was considered statistically significant.

## Results

### Study population

This pooled analysis included data from 1486 patients with efficacy data for the outcomes assessed (585 in the placebo group, 682 in the rupatadine 10 mg group, and 219 in the rupatadine 20 mg group). Mean age in the overall population was 32 years and there was a women predominance (60%). Symptom severity (T4NSS or T5SS) at baseline was comparable between placebo and rupatadine groups (Table 1).

**Table 1:** Clinical and demographic characteristics at baseline in AR patients

	Placebo (N = 585)	Rup 10 mg (N = 682)	Rup 20 mg (N = 219)
Age (years), mean (SD)	30.5 (12.1)	30.6 (12.4)	34.9 (13.4)
Sex (women), n (%)	351 (60)	415 (60.9)	127 (58)
Weight (Kg), mean (SD)	67.1 (14.1)	67.3 (14.3)	69.5 (14.3)
Height (cm), mean (SD)	167.3 (9.6)	166.6 (9.3)	168.8 (9.5)
T4NSS (0–12), mean (SD)	7.2 (2.0)	7.2 (2.0)	7.0 (1.6)
T5SS (0–15), mean (SD)	8.4 (2.6)	8.4 (2.5)	8.0 (1.9)

*AR* allergic rhinitis, *Rup* rupatadine, *T4NSS* Total 4 Nasal Symptom Score, *T5SS* Total 5 Symptom Score, *SD* standard deviation

### Change in symptom score

**Fig. 1:**
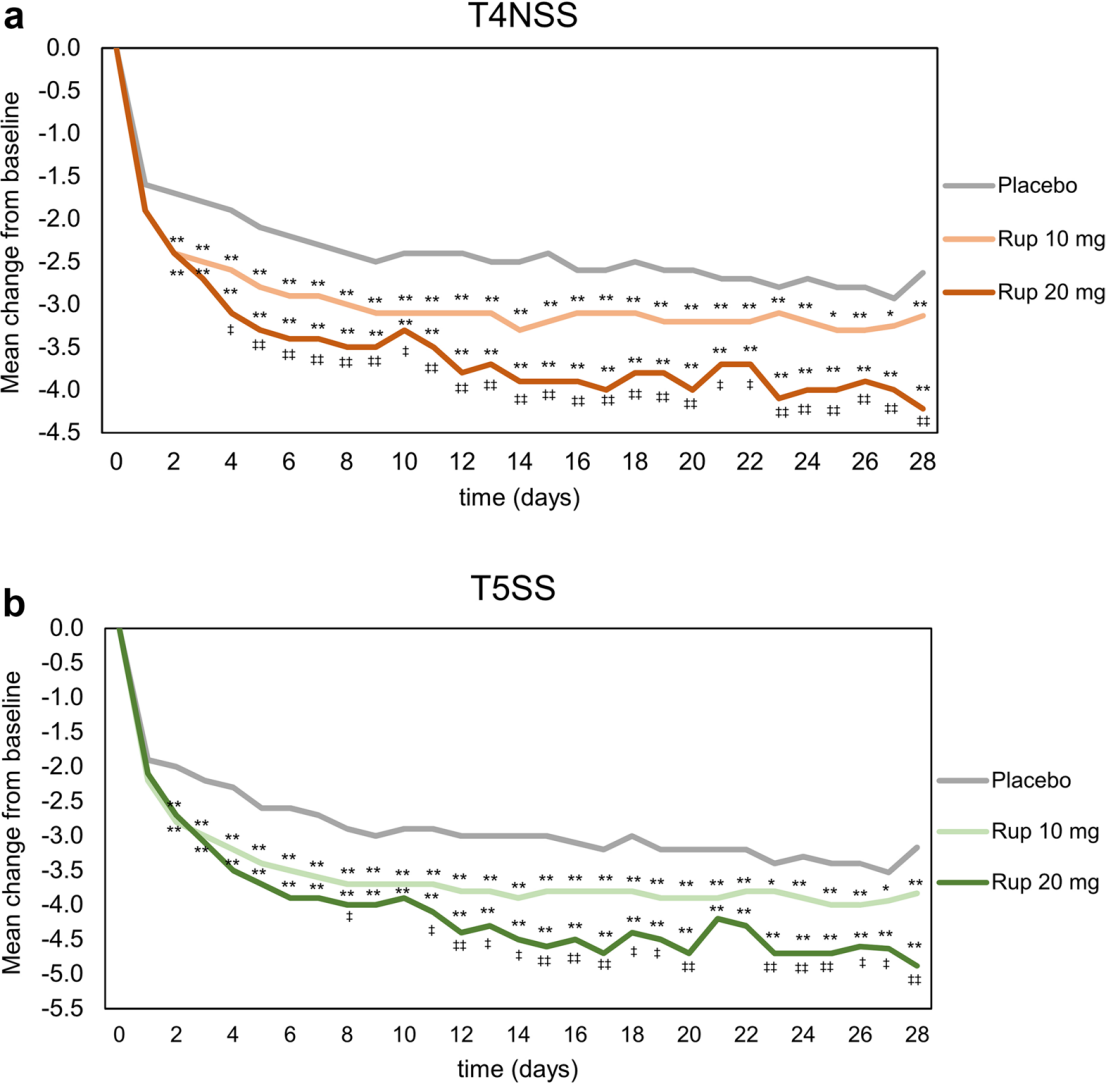
Evolution of composite symptom scores over time of treatment with rupatadine 10 mg or 20 mg, or placebo. Data are expressed as mean change from baseline over 28 days of treatment for (**a**) T4NSS, or (**b**) T5SS. Statistical significance was calculated with the Mann–Whitney test. *p < 0.05, **p < 0.01 (rupatadine groups vs placebo); ^‡^*p *< 0.05, ^‡‡^*p *< 0.01 (rupatadine 10 mg vs rupatadine 20 mg). *T4NSS* Total 4 Nasal Symptom Score, *T5SS* Total 5 Symptom Score

The T4NSS gradually decreased from baseline over 28 days in rupatadine and placebo groups. The decrease in T4NSS was significantly higher in both rupatadine groups vs the placebo group from day 2 to 28 (*p *< 0.05 rupatadine 10 mg vs placebo; *p *< 0.01 rupatadine 20 mg vs placebo). Among rupatadine groups, the rupatadine 20 mg group promoted a higher reduction compared with the rupatadine 10 mg group over the whole follow-up, with significant differences from day 4 to 28 (*p *< 0.05) (Fig. 1a).

The T5SS was also reduced in the placebo and rupatadine groups throughout 28 days of treatment, being the reduction significantly higher in rupatadine groups (*p *< 0.05 rupatadine 10 mg vs placebo; *p *< 0.01 rupatadine 20 mg vs placebo). Comparing rupatadine groups, the improvement in T5SS was significantly higher in the 20 mg group vs the 10 mg group on days 8, 11 to 20, and 23 to 28 (*p *< 0.05) (Fig. 1b).

### Responder analysis

**Fig. 2:**
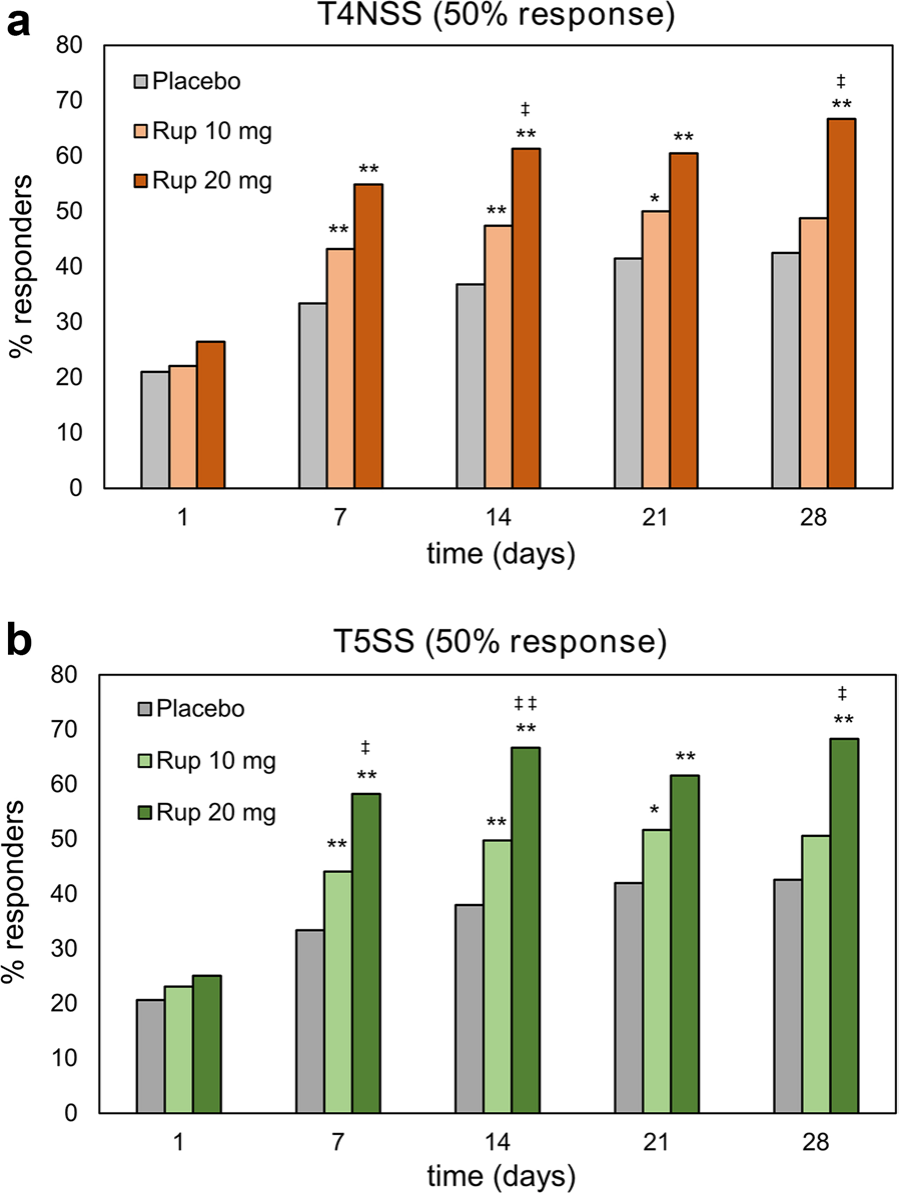
Proportion of responders achieving the 50% response for T4NSS or T5SS. Data are expressed as the percentage of patients achieving a 50% reduction in (**a**) T4NSS or (**b**) T5SS over 28 days of treatment with rupatadine 10 mg or 20 mg, or placebo. Statistical significance was determined with the Chi-square test. *p < 0.05, **p < 0.01 (rupatadine groups vs placebo); ^‡^*p *< 0.05 ^‡‡^*p *< 0.01 (rupatadine 10 mg vs rupatadine 20 mg). *T4NSS* Total 4 Nasal Symptom Score, *T5SS* Total 5 Symptom Score

The proportion of responders for the 50% response in T4NSS increased over time in rupatadine and placebo groups, being higher in rupatadine-treated patients. Differences between rupatadine and placebo groups were statistically significant on days 7, 14, 21, and 28 (*p *< 0.05 rupatadine 10 mg vs placebo; *p *< 0.01 rupatadine 20 mg vs placebo). The proportion of responders was significantly higher in the 20 mg group compared with the 10 mg group on days 14 and 28 (*p *< 0.05) (Fig. 2a). Significant differences between rupatadine and placebo groups were observed for the 50% response in T5SS from day 7 to 28 (*p *< 0.05 rupatadine 10 mg vs placebo; *p *< 0.01 rupatadine 20 mg vs placebo). The proportion of responders for the 50% response in T5SS was higher in the rupatadine 20 mg group vs the 10 mg group over the entire follow-up, and significant on days 7, 14 and 28 (*p *< 0.05) (Fig. 2b).

**Fig. 3:**
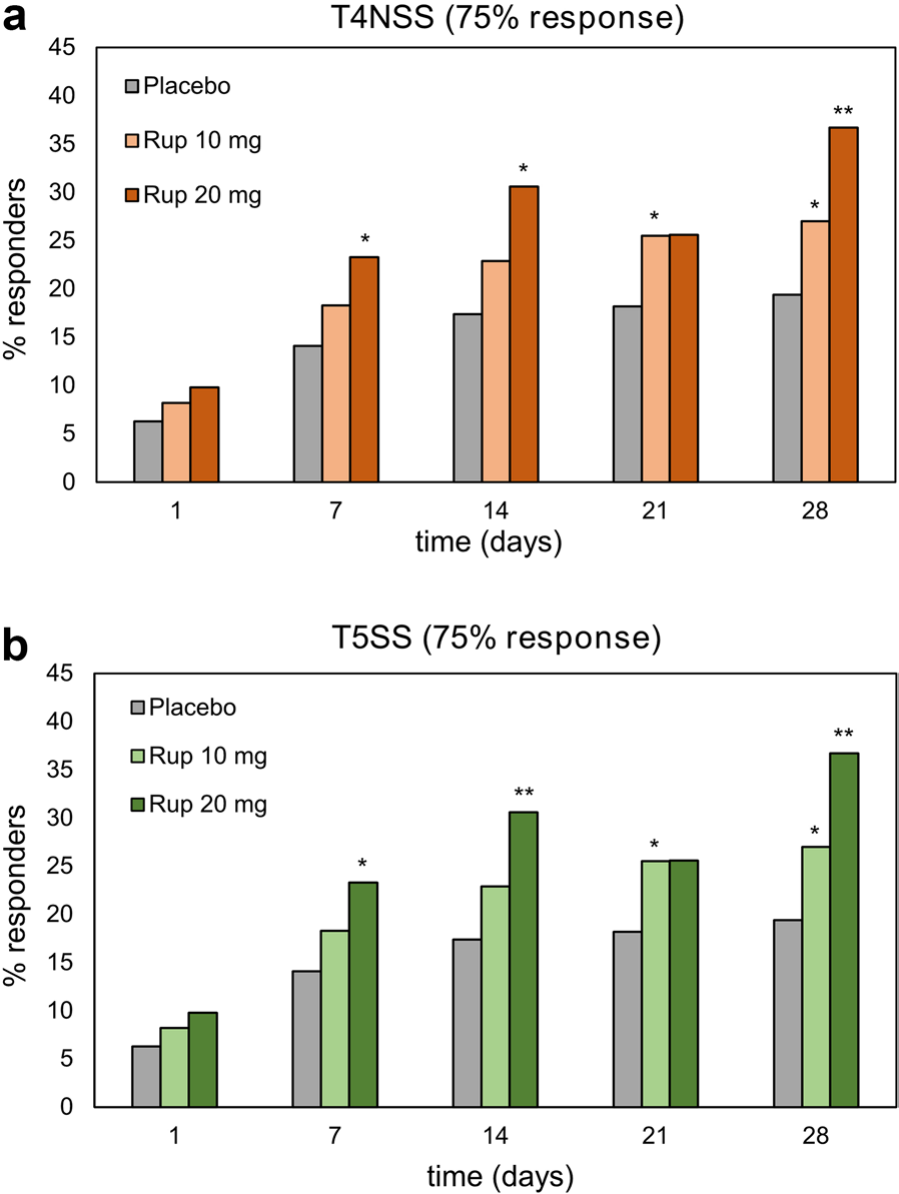
Proportion of responders achieving the 75% response in T4NSS or T5SS. Data are expressed as the percentage of patients achieving a 75% reduction in (**a**) T4NSS or (**b**) T5SS over 28 days of treatment with rupatadine 10 mg or 20 mg, or placebo. Statistical significance was determined with the Chi-square test. *p < 0.05, **p < 0.01 (rupatadine groups vs placebo). *T4NSS* Total 4 Nasal Symptom Score, *T5SS* Total 5 Symptom Score

The rate of responders for the 75% response in T4NSS generally increased in rupatadine groups, except for day 21, which showed a decrease in the rupatadine 20 mg group. In the placebo group, the proportion of responders increased from day 1 to 14 and remained stable afterwards. Patients treated with 20 mg rupatadine showed a higher proportion of responders compared with those treated with 10 mg, but differences were not statistically significant (Fig. 3a). The 75% response in T5SS showed similar results, with higher rates in rupatadine groups compared with the placebo group. Rupatadine 20 mg was associated with higher responder rates vs rupatadine 10 mg, except for day 21 when both doses showed comparable rates (Fig. 3b).

### Time to response

**Fig. 4:**
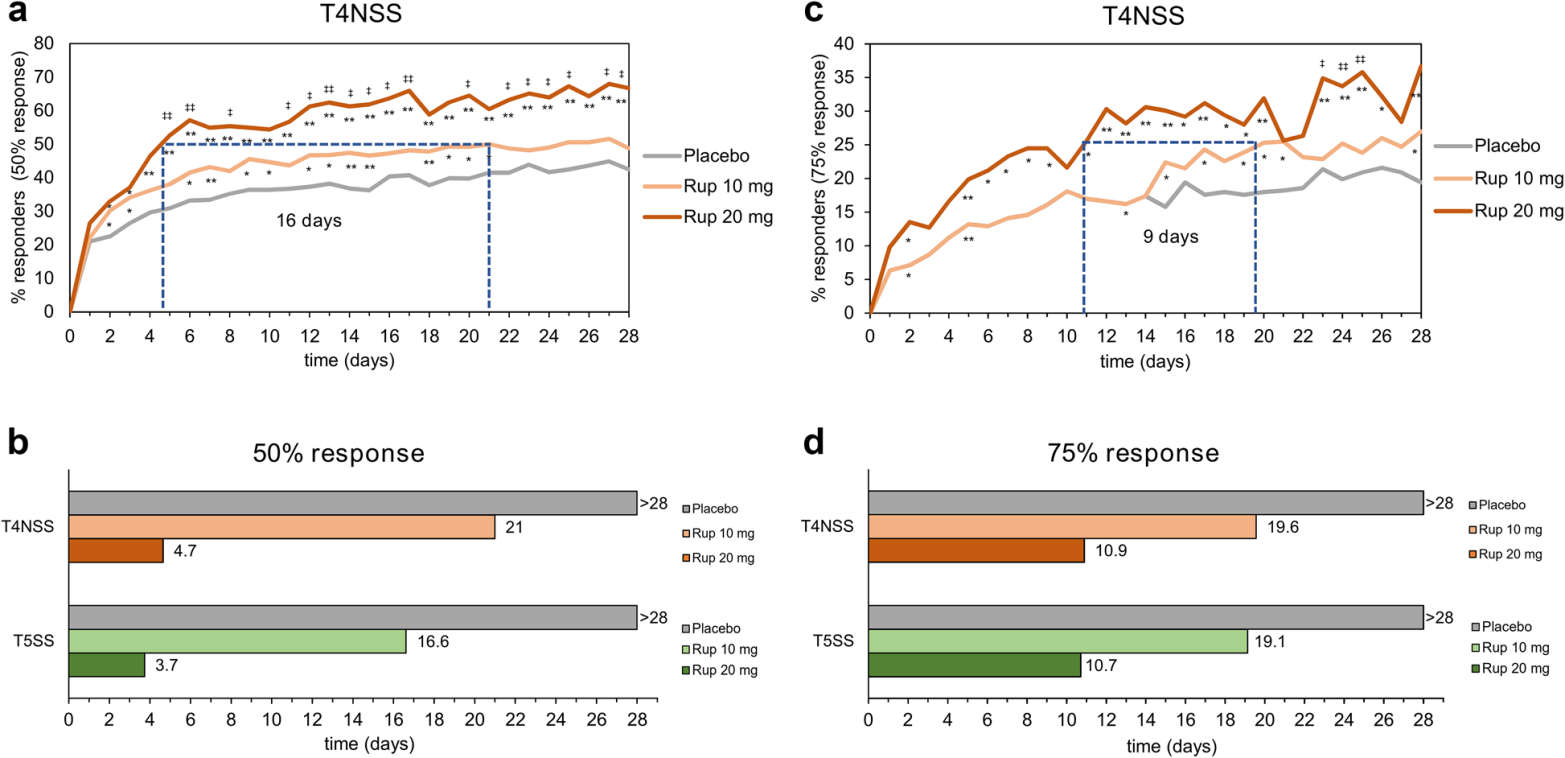
Time to achieve a 50% and 75% reduction in T4NSS or T5SS after treatment with rupatadine 10 mg or 20 mg, or placebo. **a** Proportion of responders over time for the 50% response in T4NSS. **b** Time (days) to achieve a 50% proportion of responders for the T4NSS or T5SS (50% response). **c** Proportion of responders over time for the 75% response in T4NSS. **d** Time (days) to achieve a 25% proportion of responders for the T4NSS or T5SS (75% response). *T4NSS* Total 4 Nasal Symptom Score, *T5SS* Total 5 Symptom Score

The time to achieve a 50% proportion of responders for the 50% response in T4NSS was 4.7 days in the rupatadine 20 mg group, 21 days in the rupatadine 10 mg group, and > 28 days in the placebo group (Fig. 4a, b). A 50% proportion of responders was achieved for the 50% response in T5SS after 3.7 days in the rupatadine 20 mg group, 16.6 days in the rupatadine 10 mg group, and > 28 days in the placebo group (Fig. 4b).

The time to achieve a 25% proportion of responders for the 75% response in T4NSS was 10.9 days in the rupatadine 20 mg group, 19.6 days in the rupatadine 10 mg group, and > 28 days in the placebo group (Fig. 4c, d). A 25% proportion of responders was achieved for the 75% response in T5SS after 10.7 days in the rupatadine 20 mg group, 19.1 days in the rupatadine 10 mg group, and > 28 days in the placebo group (Fig. 4d).

Taken together, the time to response was shorter in the rupatadine 20 mg group vs the 10 mg group for the T4NSS (by 16 days for the 50% response and 9 days for the 75% response) and the T5SS (by 13 days for the 50% response and 8 days for the 75% response).

## Discussion

Rupatadine promoted a rapid and sustained improvement of AR symptoms in patients with moderate-severe PAR in this pooled analysis of data. Among rupatadine groups, the response to the 20 mg dosage was significantly better than to rupatadine 10 mg in patients with moderate-severe PAR, providing higher and faster responses. This responder analysis supports and adds new evidence on the efficacy of rupatadine in moderate-severe PAR patients. The analysis included data from a representative sample of 1486 patients with PAR from different countries. In agreement with previous studies [29–32], mean age in the overall population was 32 years, with a female predominance (60%). At baseline, mean T4NSS was 7.1 (out of 12) and mean T5SS was 8.3 (out of 15) in the pooled sample, which limits the conclusions of the study to patients with moderate-severe PAR, who are more likely to visit the specialist and receive appropriate care [33].

Rupatadine provided an early and sustained response in symptom improvement. Nasal symptoms (T4NSS) progressively improved in rupatadine and placebo groups throughout the 28-day treatment period, with higher improvements in rupatadine groups. The relief in nasal symptoms was already evident after the first day of treatment (25.6% reduction with rupatadine 10 mg and 26.5% with 20 mg), as previously observed [34]. The rapid improvement is in keeping with previous pharmacokinetic studies showing the fast absorption of oral rupatadine, reaching maximum concentrations after 30–45 min [35]. Although the T4NSS was reduced during the entire follow-up in rupatadine groups, the reductions were less prominent from day 10 onwards in the rupatadine 10 mg group and from day 14 in the rupatadine 20 mg group, indicating that this higher dose presents a more enduring and sustained efficacy. At the end of follow-up (day 28), the T4NSS was reduced by 46% and 58.6% in rupatadine 10 mg and 20 mg, representing a 6.8% and 19.5% higher reduction vs placebo, respectively. The percentage of improvement from baseline to day 28 in T4NSS observed in previous studies with other antihistamines was 32% for cetirizine, 34.7% for bilastine, and 37.9% for desloratadine [24, 31, 36]. The fact that the improvement in T4NSS with 20 mg rupatadine did not *plateau* at the end of follow-up reinforces the interest in studying the efficacy of this antihistamine and PAF antagonist for extended periods [25, 37].

The total symptom score (T5SS), which also considers ocular itching, showed a similar trend than the T4NSS, but differences between groups were less pronounced. This effect could indicate that the impact of rupatadine is more evident on nasal symptoms than on ocular itching and that the ocular component is less clinically relevant in PAR. In this regard, previous studies showed that rhinorrhea and sneezing were the main symptoms that improved with rupatadine compared to placebo [22, 23]. Rupatadine groups showed a comparable trend between days 1 and 10, with remarkably greater improvements in the rupatadine 20 mg group from day 10 onwards. These results point to a similar early effect of both dosages for the combination of nasal symptoms and ocular itching, but a more sustained response with 20 mg.

Few studies have assessed the clinical relevance of an AR treatment in terms of responders for the 50% or 75% response [27, 38] and, to our knowledge, this is the first conducted on patients with PAR. Regulatory guidelines encourage the determination of whether differences between active treatments and placebo are not only statistically significant but also clinically relevant. The EMA guideline on the treatment of allergic rhinoconjunctivitis recommends analysing the proportion of responders for the 50% response [26]. Following this criterion, we observed a progressive increase of responders with rupatadine treatment, which was also noted with the stricter cut-off of 75%. Although the placebo effect is unequivocal as previously shown [22–25, 34], it is important to highlight that the proportion of placebo-treated patients who reached the 50% and 75% response slightly increased from day 14 onwards, contrasting with the steady increase in responders among rupatadine-treated patients. Whether this higher rate of responders in rupatadine groups would translate into an improvement in quality of life requires further investigation. In the study of Fantin et al. rupatadine-treated patients showed a better perception of quality of life as compared with placebo-treated patients after 3 months [25].

Given the impact of PAR on daily life activities and quality of life, it is of capital importance to seek treatments providing rapid and sustained symptom relief. For this reason, we compared the time to achieve a proportion of responders in T4NSS and T5SS for both response cut-offs. Importantly, the rupatadine 20 mg group achieved a 50% proportion of responders for the T4NSS and T5SS after 4.7 and 3.7 days, respectively, contrasting with the 21 and 16.6 days required with rupatadine 10 mg. This implies that doubling the dose of rupatadine may lead to faster achievement of symptom relief. In spite of the greater effectiveness with the 20 mg dosage, rupatadine is mostly authorised at 10 mg for mild-moderate AR patients. Furthermore, 20 mg rupatadine once daily is authorised for AR Japanese patients whose nasal symptoms cannot be effectively controlled within 1 to 2 weeks of rupatadine 10 mg therapy, which is in agreement with the results observed in this pooled analysis.

Comparing the results obtained in the pooled analysis of patients with SAR [27], the reduction in symptom scores from baseline to day 14 was systematically higher in patients with SAR vs those with PAR, although differences vs placebo were similar. We observed higher rates of responders in patients with SAR compared with those with PAR. Interestingly, differences between rupatadine groups in the time to response were higher in patients with PAR compared with those with SAR. Although this comparison is indirect and requires further confirmation, it could imply that increasing the dose of rupatadine to 20 mg may have more significant benefits in patients affected by PAR, although higher improvements are reached in those suffering from SAR. These results can be explained considering that patients with SAR tend to experience more acute symptoms, allowing more room for improvement.

This study presents some limitations. First, the pooled analysis did not include safety assessments comparing the risk/benefit ratio for each dose. In this regard, previous safety data showed that somnolence is more prevalent with 20 mg than with 10 mg rupatadine. Second, we did not evaluate the improvement in individual nasal symptoms, which could reveal the main contribution of rupatadine to patients’ improvement. Third, since T4NSS ≥ 5 was an inclusion criterion in most of the studies included [22–24], this study mainly comprises patients with moderate-severe PAR and is not extendable to patients with mild severity who frequently self-medicate and are not often diagnosed. Fourth, almost all studies included did not use the newer ARIA classification (intermittent and persistent AR), so our analyses focus on patients with PAR, a term that is not interchangeable with persistent AR. Last, comparisons between rupatadine groups should be analysed cautiously considering that the rupatadine 20 mg included fewer patients than the 10 mg group.

In contrast, this pooled analysis is the first in assessing the effectiveness of rupatadine in terms of responders and time to response in PAR patients. These results support those recently published in patients with SAR [27] and raise knowledge in PAR response to rupatadine. The promising and robust results obtained in a large and representative population of patients with PAR may help guide treatment decisions for PAR, a condition particularly challenging to manage. Studies performing head-to-head comparisons between antihistamines for the response criteria defined here and assessing the impact on quality of life warrant further investigation.

## Conclusion

Rupatadine is effective in reducing nasal symptoms and ocular itching, showing a dose-related effect, with a higher proportion of responders, faster onset of action and more sustained effects with 20 mg rupatadine vs 10 mg rupatadine or placebo in PAR patients. This faster and stronger effect of rupatadine 20 mg may suggest its use in patients with severe PAR or not responding to the standard dose.

## Supplementary information

**Additional file 1.** Additional tables.

## Data Availability

The datasets used and/or analyzed during the current study are available from the corresponding author on reasonable request.

## Abbreviations

AIT: Allergen immunotherapy
AR: Allergic rhinitis
ARIA: Allergic Rhinitis and its Impact on Asthma
CRS: Chronic rhinosinusitis
EMA: European Medicines Agency
PAF: Platelet-activating factor
PAR: Perennial allergic rhinitis
SAR: Seasonal allergic rhinitis
SD: Standard deviation
T4NSS: Total 4 Nasal Symptom Score
T5SS: Total 5 Symptom Score
