# Supplementary material for: Higher efficacy of rupatadine 20 mg and 10 mg versus placebo in patients with perennial allergic rhinitis: a pooled responder analysis
Source: Allergy Asthma Clin Immunol. 2020 Apr 23;16:29. doi: 10.1186/s13223-020-00425-1 (PMC7181536; doi:10.1186/s13223-020-00425-1)
Supplement: Supplementary file 1 — Additional file 1. Additional tables. [file 13223_2020_425_MOESM1_ESM.docx]

**ADDITIONAL FILES**

**Table S1.** Clinical studies included in the pooled analysis of patients with PAR.

| **Study** | **Design** | **Comparator** | **No. patients**  **Intention-to-treat** | **Primary**  **endpoint** | **Symptoms** | **Duration** |
| --- | --- | --- | --- | --- | --- | --- |
| Kowalski *et al.* [23] | Multicentre, randomised, double-blind, parallel-group, placebo-controlled study | RUP 10, 20mg  LOR 10mg  PL | 283 | T5SS | Running nose  Itchy nose  Nasal congestion  Sneezing  Ocular itching | 28 days |
| Molina *et al.* [22] | Multicentre, randomised, double-blind, parallel-group, placebo-controlled study | RUP 10mg  EBA 10mg  PL | 219 | T5SS | Running nose  Itchy nose  Nasal congestion  Sneezing  Ocular itching | 28 days |
| Marmouz *et al*. [24] | Multicentre, randomised, double-blind, parallel-group, placebo-controlled study | RUP 10, 20mg  CET 10mg  PL | 269 | T5SS | Running nose  Itchy nose  Nasal congestion  Sneezing  Ocular itching | 28 days |
| IC01RUPIV02 study code * | Multicentre, randomised, double-blind, parallel-group, placebo-controlled study | RUP 10mg  PL | 324 | T5SS | Running nose  Itchy nose  Nasal congestion  Sneezing  Ocular itching | 28 days |
| Fantin *et al*. [25] | Multicentre, randomised, double-blind, parallel-group, placebo-controlled study | RUP 10mg  CET 10mg  PL | 543 | T6SS | Running nose  Itchy nose  Nasal congestion  Sneezing  Ocular itching  Ocular redness | 28 days** |
| URFC97II_01 study code* | Multicentre, randomised, dose-ranging, double-blind, parallel-group, placebo-controlled study | RUP 10, 20mg  PL | 153 | T5SS | Running nose  Itchy nose  Nasal obstruction  Sneezing  Itchy eyes | 28 days |

RUP, rupatadine; PL, placebo; LOR, loratadine; EBA, ebastine; CET, cetirizine; T5SS, total 5 symptom score; T6SS, total 6 symptom score; PAR, perennial allergic rhinitis.

* Data on file.

** Treatment study duration was 90 days; for this analysis, data from the first 28 days were considered.

**Table S2.** Effects of rupatadine treatment on T4NSS and T5SS.

|  | **Placebo**  **(N = 585)** | **Rup 10mg**  **(N = 682)** | **Rup 20mg**  **(N = 219)** | ***p*^a^** |
| --- | --- | --- | --- | --- |
| **T4NSS (0-12)** |  |  |  |  |
| Day 14 |  |  |  |  |
| Mean score (SD) | 4.6 (2.8) | 3.9 (2.7)* | 3.3 (2.2)*^‡^ | <0.001 |
| % change^b^ | 35.1% | 45.1% | 53.6% |  |
| Day 28 |  |  |  |  |
| Mean score (SD) | 4.4 (2.9) | 3.9 (2.9)* | 2.9 (2.1)*^‡^ | <0.001 |
| % change | 39.2% | 46.0% | 58.6% |  |
| **T5SS (0-15)** |  |  |  |  |
| Day 14 |  |  |  |  |
| Mean score (SD) | 5.7 (3.3) | 4.8 (3.0)* | 4.1 (2.7)*^‡^ | <0.001 |
| % change | 36.2% | 46.3% | 54.9% |  |
| Day 28 |  |  |  |  |
| Mean score (SD) | 5.0 (3.4) | 4.5 (3.4)* | 3.3 (2.3)*^‡^ | <0.001 |
| % change | 39.9% | 47.1% | 58.4% |  |

^a^ Statistical significance was determined using the non-parametric Kruskal-Wallis test.

^b^% change from baseline

* indicates *p*<0.05 (rupatadine groups vs placebo) calculated with the Mann-Whitney test.

‡ indicates *p*<0.05 (rupatadine 10mg vs rupatadine 20mg) calculated with the Mann-Whitney test.

Rup, Rupatadine; T4NSS, Total 4 Nasal Symptom Score; T5SS, Total 5 Symptom Score.
